# Process Evaluation of Skilled Delivery Service in Hadiya Zone in Southern Nations, Nationalities, and Peoples Region, Ethiopia

**DOI:** 10.1155/2020/4717520

**Published:** 2020-02-05

**Authors:** Tagesse Sedoro, Tekle Ejajo, Lonsako Abute, Tirulo Kedir, Belay Erchafo

**Affiliations:** ^1^Monitoring Evaluation Specialist, Department of Public Health, Wachemo University, Hosanna, Ethiopia; ^2^Health Service Management Specialists, Department of Public Health, Wachemo University, Hosanna, Ethiopia; ^3^Health Education and Behavioral Science Specialist, Department of Public Health, Wachemo University, Hosanna, Ethiopia; ^4^Environmental Health Science Specialist, Department of Public Health, Wachemo University, Hosanna, Ethiopia

## Abstract

Pregnancy-related death is a cause for maternal and newborn mortality and morbidity as well as an obstacle for economic growth. Three-quarters of mothers' lives can be saved if women have access to a skilled health worker at delivery and emergency obstetric care. This evaluation was conducted to assess skilled delivery service implementation level by using three dimensions (availability, compliance, and acceptability) and identify major contributing issues for underutilization of the service. The evaluation design is cross-sectional. The study included 846 mothers who gave birth in Hadiya zone within one year prior to study period, using one year delivery records. Epi Info 3.5.3 and SPSS version 16 were employed for data analysis. Based on selected indicators, resource availability was inadequate for health facilities, human resource medical equipment, and rooms. On the compliance dimension, skilled delivery service coverage (34.8%), active management of third stage labor (32.7%), and health information at discharge and in postnatal care (PNC) visit (7.1%) critically complied with or poorly agreed to the guidelines and targets. Regarding skilled delivery service acceptability, welcoming, privacy keeping, reassurance during labor pain, follow-up, baby care, comfortability (rooms, beds, and clothing), cost of service, and episiotomy (without local anesthesia) were not acceptable.

## 1. Background

Process evaluation is an evaluation that assesses and describes program inputs activities and outputs in order to improve delivery service (e.g., identify obstacles and adjust activities) [1].

Skilled delivery service or care refers to the service provided to a woman and her newborn during pregnancy, childbirth, and immediately after birth by an accredited and competent health care provider who has at her/his disposal the necessary equipment and the support of a functioning health system, including transport and referral facilities for emergency obstetric care [2].

The deaths from the complications of pregnancy are estimated that 127 000 women (25%) die due to hemorrhage, 76 000 (15%) due to sepsis, 65 000 (12%) due to hypertensive disorders of pregnancy, 38 000 (8%) due to obstructed labor, and almost 67 000 (13%) due to abortion. Maternal deaths occur due to the same complications throughout the developing world, yet the technology to prevent them exists. Maternal death is only the last chapter in a history that starts much earlier in a woman's life. Among maternal deaths, 99% are in developing countries and a high proportion of these deaths occur in sub-Saharan Africa [3–5].

Every year at least 7 million women who survive childbirth suffer serious health problems and further 50 million women suffer adverse health consequences after childbirth such as anemia or reproductive tract infections or lifelong disabilities, such as obstetric fistulae [4, 5].

In developing countries, 1 in 16 may die of pregnancy-related complications compared to 1 in 2800 in developed countries [3]. The complications that affect women during pregnancy and childbirth affect the fetus as well. Around 8.1 million children die each year and many of these deaths are a direct consequence of poorly managed pregnancies and deliveries. Millions of infants survive but with a degree of damage that renders them physically or mentally disabled throughout their lives. Around the world newborn babies die/damage due to similar causes; these include birth asphyxia, trauma, or infections [6].

To achieve millennium development goal, that is, 75% reduction of maternal mortality by 2015, the United Nations General Assembly set the target for all countries to “continue their efforts so that globally, by 2005; 80% of all births should be assisted by skilled attendants, by 2010; 85%, and by 2015; 90%.” Currently, however, the global percent is 65% and in Africa 46%. This indicates that the world and Africa are lagging behind the MDG target for skilled attendance at birth [2, 7, 8].

In Ethiopia, the maternal mortality ratio is 676/100,000 life birth. In other words, for every 1,000 live births, about seven women (6.76) die during pregnancy, during childbirth, or within two months of childbirth. The lifetime risk of maternal death (0.036) indicates that about 4% of women die during pregnancy, during childbirth, or within two months of childbirth [9].

According to Ethiopian Demographic and Health Survey (EDHS) 2011, in spite of the improvement (6% to 10%) in SBA, Ethiopia is still lagging behind from the target (85%, to be achieved by 2010) set by the United Nations General Assembly to achieve the MDG 5 [7, 9].

Ethiopia has a shortage of health professionals at all levels, including doctors, nurses, and midwives and poor clinical governance processes to monitor standards of health care [10].

In general skilled delivery service utilization level is low in Ethiopia, in Southern Nations, Nationalities, and Peoples' Region (SNNPR); the same is true for Hadiya zone as it has been seen from the administrative report of 2005 Ethiopian financial year (EFY). In addition, skilled birth attendance (SBA) achievement is low in comparison with other family health services (SBA = 24%, ANC = 59%, FP = 43%) [9, 11].

Although evaluation, especially process evaluation, has a pivotal role in gap identification, information provision for decision making of skilled delivery service is not still evaluated in this specific zone. This, in turn, hampers the right decision making capacity of the managers and other stakeholders as well as program improvement [12].

Conducting process evaluation might examine whether the activities are taking place, who is conducting the activities, who is reached through the activities, and whether sufficient inputs have been allocated. Process evaluation is important to distinguish the causes of poor program performance, whether the program was a bad idea or a good idea that could not reach the standard for implementation that you set, and how it actually operates on a daily basis. In all cases, process evaluations measure whether actual program performance was faithful to some initial plan [13].

It is obvious that to improve certain service utilization, decision making is the issue of the priority. Again to make the right decisions, information is very crucial. So this evaluation is expected to assess specific dimensions of program implementation by using different indicators, providing valuable information for decision making and program improvement. In short, the rationale of this evaluation is the interest of the stakeholders, Hadiya Zone Health Department to improve delivery service utilization. In addition, this particular zone lacks any prior evaluation on the implementation level of the skilled delivery service program so that this evaluation will be used as a baseline for future program evaluations and other researches.

## 2. Methods and Materials

### 2.1. Study Area and Period

The study was conducted in Hadiya zone, which is located in southern Ethiopia, 230 km from Addis Ababa. This skilled delivery service program evaluation was conducted from April 1 to April 30 2014, by focusing on its process through a formative approach, by using dimensions, such as availability, compliance, and acceptability. The evaluation design was cross-sectional.

### 2.2. Sample Size Determination and Sampling Technique

#### 2.2.1. Sample Size Determination

The required sample size of the study population is calculated using the formula for single population proportion according to the following assumption, where *n* = the required sample size, *z* = standard error corresponding to 95% confidence level = 1.96, *p* = the proportion of women attending institutional delivery, and since there were no previous similar study conducted on acceptability in the study area so, I will use *p*=0.5 to yield maximum sample size, *d* = the margin of error = 5%. Factor two is used for the design effect.

The required sample size was determined by using one proportion formula:

(1) $$\begin{array}{c} n=\frac{Z^{2}p\left(1-p\right)}{w^{2}},\\ n=\frac{1.96^{2}*0.5\left(1-0.5\right)}{0.0025}=384.16. \end{array}$$

Since the sampling technique is multistage sampling, *two* is considered for *design effect* and sample size = 2 ∗ 384.16 = 768.32 and the nonresponse rate of 10%(77); the total sample size is 846 women who gave birth within the last year preceding the evaluation.

#### 2.2.2. Sampling Technique and Procedures: Stratified Multistage Random Sampling

According to EDHS 2011, urban births are notably higher to be delivered in a health facility than rural births (50% vs 4 %) [10]. The study area was stratified into urban and rural. There are ten rural and one town administration. Due to resources and cost reasons from rural districts, two were selected randomly by lottery method. The Capital Town, Hossana, was purposefully included in the sampling to represent the urban communities. From these three districts, six kebeles were randomly selected. From this, a total of six kebeles, sampling frame was prepared for mothers who gave birth during the last year. The allocated sample size for urban and rural stratum was obtained using probability proportional allocation to the size (PPS) of mothers found in each selected kebeles. Finally 846 mothers were randomly selected and interviewed.

*Document review*. Resource availability of skilled birth attendants and health facilities for delivery service were reviewed from Hadiya zone 2006 Ethical Administrative Report document.

### 2.3. Data Collection and Quality Control

The document review checklists and structured and semistructured questionnaires were translated into Amharic language and again back to English by another person who has the same level of language capacity on both languages as to ensure that the meaning is the same, culturally applicable, and consistent.

The data collectors were experienced in health data collection, with a minimum qualification of diploma in nursing.

The number of data collectors was twelve with six supervisors. One day training was conducted before data collection for all data collectors and supervisors. The training was conducted in the form of a thorough discussion, by focusing on the general objectives of the study, discussing the contents of the data collection tools one by one and the type of information needed to be handled and how to handle any possible questions as well as problems that may arise during data collection and discussions on how to maintain confidentiality and privacy. Data collection was started immediately after training. The collected data was checked daily and supervision also took place throughout the data collection by the principal investigator in addition to supervisors.

### 2.4. Data Management and Analysis

The quantitative data from the mothers' interview was cleaned, edited, and entered into Epi Info 3.5.3; then the data was exported to SPSS version 16; then the data were analyzed, interpreted, and presented. After analysis, the data were described and presented using tables and graphs.

### 2.5. Ethical Issues

Ethical clearance was taken from the Research and Community Service Vice President Office of the Wachemo University and submitted to Hadiya Zone Health Department and a similar letter was obtained from the Zonal Health Department to selected districts, then to health facility and kebele. During data collection, the participants were informed and verbal consent was obtained, following an explanation about the purpose of the interview and no name of any individual was requested or registered. If no consent was gained from the 1^st^ mother, then the next mother was considered. The study subject had full right to refuse totally and to withdraw at any time without precondition.

## 3. Results and Discussions

### 3.1. Sociodemographic Characteristics of Respondents

A total of 846 women who gave birth one year prior to the data collection period were interviewed. The response rate was 100%.

Two hundred thirty-four (86.3%) of the women were in the age group of 20–34 years with a mean age of 26.05 ± 4.89 SD and the majority (73.1%) were attended less than grade ten.

The majority 759 (89.7%) of respondents were Hadiya in ethnicity, 620 housewives (73.2%) in occupation, 736 protestant in religion (86.9%), and 829 married, for marital status (97.9%).

Five hundred twenty (61.4%) of the respondents had monthly income less than 600 birrs and 326 (38.5%) reported equal or greater than 600 birrs per month. The average monthly income was 1206.39 ± 1343.65 birrs SD (Table 1).

### 3.2. Obstetric Characteristics of the Respondents

Two (0.2%) of the women were pregnant before the age of 20 years and 844 (99.8%) were above 20 years of age when they were first pregnant with a mean age at first pregnancy of 22.1 ± 3.0 SD. Out of all the respondents, 230 (27.2%) women had five or more children and 253 (29.9%) of women had five or more pregnancies. Regarding the child survival issue, those who had an abortion history were131 (15.5%), stillbirth 90(10.6%), neonatal death 12 (1.4%), and infant death 29 (3.4%) (Table 2).

### 3.3. Maternal Health Care Utilization

Among delivery service users, some (15.3%) had not used health facilities for ANC service and among ANC service users most (74.9%) of them had not been informed about health facility delivery service (Table 3).

### 3.4. Availability

According to the selected indicators, the level of human resource availability was relatively adequate (100%) with regard to trained staff in comprehensive emergency obstetric care (cesarean section), anesthetists (>100%), and nurses (>100%) but inadequate with regard to all type of physician (1 : 61914 < 1 : 10000), all types of skilled birth attendants (23 : 27815 < 23 : 10 000), health officers (1 : 17392 < 1 : 10000), and midwives (1 : 12687 < 1 : 6759) in comparison with the standards of HSDP IV [14].

The availability of facilities was inadequate for health center (98.9%) and adequate for blood bank but there was a shortage of functional general hospitals (1 : 1547848 < 1 : 1 million) [14] (Table 4).

### 3.5. Compliance

In the compliance dimension, the average result (46.9%) of all selected indicators shows that the program compliance level was Critical; in comparison with the United Nation and Ethiopian national guidelines and targets, there were some least complied indicators. These were deliveries conducted by active management (32.7% vs. all or 100%), pregnant women who have got skilled delivery service (34.8% vs. 62% or 85%), and mothers who got health information at discharge on PNC (74.9% vs. all or 100%) [7, 14, 15] (Table 5).

To achieve the millennium development goal (75% reduction of maternal mortality by 2015), the United Nations General Assembly set the target for all countries to achieve skilled delivery service coverage of 85% by 2010, but zonal coverage was 294 (34.8%). This indicates that Hadiya zone is lagging behind the UN and national target (62%) for skilled attendance at birth [7, 14, 15].

As the standard of safe motherhood, the mother should get PNC at least three times, but most 273 (92.9%) of mothers were not informed to do so at discharge. This was two times higher in comparison with that of countdown countries (45%) information on postnatal care for women [16].

On the cesarean section, the proportions (7.1%) of mothers operated were within the range of the UN recommendation level (5–15%) [14, 15].

### 3.6. Acceptability

From the view point of the selected indicators, the way of delivery service program implementation in the zone health facilities was adequately accepted by the majority, but from some client suggestion, welcoming, privacy keeping, reassurance during labor, follow-up, baby care, comfortability (rooms, beds, and clothing), cost of service, and episiotomy (without local anesthesia) were suggested to be improved. Hospital delivery service cost was as follow in addition to transport and other costs: spontaneous vaginal delivery: 40 birrs, assisted delivery: 75 birrs, retained placenta removal: 100 birrs, cesarean section: (c/s)1000 to 1500 (this cost is not for c/s only but also for food, for drugs, and other medical supplies from private pharmacies). These cost details indicate that hospital delivery service was costly (Table 6).

Service cost was suggested to be free or minimized by clients in all health facilities including hospital. So service cost might be one of the issues to be dealt with to improve delivery service. In Ghana removing service fee or free care at birth resulted in a sharp increase of births in health facilities [17].

*Limitation*: recall bias and misreporting of events were likely in this study as it requires the mothers to remember events that happen before sometimes to one year. The study employed few selected indicators for this skilled delivery service program evaluation because of time and cost reason being also additional limitations.

## 4. Conclusions

In availability dimension evaluation, there was a gap in human resources (all type of physicians, health officers, and midwives), medical equipment, rooms, and health facilities (hospital).

The activities such as active management of the 3rd stage of labor and mothers who received health information at discharge on postnatal care were critically (<50%) compliant.

In acceptability dimension, the cost of delivery service, the birth attendant's response to their questions during labor, birth attendants' help and support during labor, and welcoming of the health facilities were less acceptable.

## Figures and Tables

**Table 1:** Sociodemographic characteristics of respondents in Hadiya zone, SNNPR, Ethiopia, April 2014.

Variable	Categories	Number (*n* = 846)	Percentage
Age	15–19 years	5	0.6
	20–34 years	735	86.9
	35–49 years	106	12.5
	Mean ± SD		29.4 ± 4.36

Educational status	Illiterate	151	17.8
	1–4	185	21.9
	5–8	261	30.9
	9–10	76	8.9
	10 + 1 to 10 + 3	153	18.1
	Others	20	2.3

Ethnicity	Hadiya	759	89.7
	Kambata	47	5.5
	Gurage	13	1.5
	Silte	11	1.3
	Amhara	10	1.1
	Others	6	0.7

Occupation	Housewife	617	72.9
	Government employee	144	17.0
	Merchant	64	4.5
	Student	14	1.6
	Others	6	0.7

Religion	Protestant	732	86.5
	Muslim	75	8.9
	Orthodox	35	4.1
	Others	4	0.5

Marital status	Married	840	99.3
	Others	6	0.7

Month income	<600 birr	308	36.4
	≥600	536	63.6
	Mean ± SD		1206.39 ± 1343.65

**Table 2:** Obstetric characteristics of respondents in Hadiya zone, SNNPR, April 2014.

Variable	Categories	Number (*n* = 846)	Percentage
Age at 1^st^ pregnancy	<20	2	0.2
	≥20	844	99.8
	Mean ± SD		22.1 ± 3.0

Gravida	<5	593	70.1
	≥5	253	29.9

Parity	<5	616	72.8
	≥5	230	27.2

Number of live births	0	7	0.8
	1	163	19.2
	2–4	638	75.4
	≥5	208	24.6

Ever had abortion	Yes	131	15.5
	No	715	84.5

Ever had stillbirth	Yes	90	10.6
	No	756	89.4

Ever had neonatal death	Yes	12	1.4
	No	834	98.6

Ever had infant death	Yes	29	3.4
	No	817	96.6

**Table 3:** Maternal health care utilization among respondents in Hadiya zone, SNNPR, April 2014.

Health service	Category	Number	%
ANC received		(*n* = 846)	
	Yes	717	84.7
	No	129	15..3

Delivery service	Health facility delivery	294	34.8
	Home delivery	552	65.2

Health education: At ANC visit		(*n* = 717)	
	Yes	671	93.6
	No	46	6.4

Health education: About health facility delivery		(*n* = 671)	
	Yes	503	74.9
	No	168	25.1

**Table 4:** Matrix of analysis and judgment criteria for availability dimension indicators, in Hadiya zone, Ethiopia, April 2014.

S. no.	Indicator	Resource at hand	Expected result of indicator	Gained result of indicator	%	Judgment criteria	Judgment of indicator
1	Presence of at least 4 trained staff in comprehensive emergency obstetric care (cesarean section)	4	10	10	100	>85% adequate, 70–85% acceptable, 50–69.9% partially equipped, <50% critical	Adequate
2	Presence of at least 3 anesthetist	5	7	7	166.7		Adequate
3	Presence of at least one functional blood bank	1	8	8	100		Adequate
4	Proportion of all physician to population(target 1 : 10000)	25(1 : 61914)	9	1.5	16.7	1 : 10000 adequate, (≥85%), >1 : 10000, inadequate (<85%)	Inadequate
5	Proportion of health officers to population(target 1 : 10000)	89(1 : 17392)	8.5	4.9	57.6		Inadequate
6	Proportion of midwives to population (target 1 : 6759)	122(1 : 12687)	6	4.5	75	1 : 6759 adequate, (≥85%), >1 : 6759 inadequate (<85%)	Inadequate
7	Proportion of all type nurses to population (target 1 : 4725)	1041(1 : 1487)	6	19	316.7	1 : 4725 adequate, (≥85%), >1 : 4725 inadequate (<85%)	Adequate
8	Proportion of functional general hospital to population(target 1 : 1 million)	1(1 : 1547848)	10	6.5	65	1 : 1000000 adequate, (≥85%), >1 : 1000000 inadequate (<85%)	Inadequate
9	Proportion of functional health center to population (target 1 : 25,000 population)	61(1 : 25375)	9	8.9	98.9	1 : 25000 adequate, (≥85%), >1 : 25000 inadequate (<85%)	Inadequate
10	Proportion of SBA to population (WHO estimate ≥23 : 10 000 population) to achieve MDG	1277(23 : 27815)	10	3.6	35.9	≥23 : 10 000 population adequate, (≥85%), <23 : 10 000 population inadequate (<85%)	Inadequate
	*Total availability score and judgment*		*83.5*	*73.9*	*88.5*	*—*	*Adequate*

**Table 5:** Matrix of analysis and judgment criteria for compliance dimension indicators, Hadiya zone, Ethiopia, April 2014.

S.no.	Indicator	Result		Expected weight of indicator	Gained wt. of indicator	%	Judgment criteria and judgment	Judgment of indicator
		Num	%					
1	% of pregnant mothers who got health information on skilled delivery service (*n* = 671)	503	74.9	5	3.7	74.9	>85% adequate 70–85% acceptable 50–69.9% partial <50% critical	Acceptable
2	% of pregnant women that has got skilled delivery service (*n* = 846)	294	34.8	6	2.1	34.8		Critical
3	% of delivery conducted by active management of third stage (*n* = 294)	96	32.7	7	2.3	32.7		Critical
4	% of mothers who got health information at discharge on PNC visit (*n* = 294)	21	7.1	5	0.4	7.1		Critical
5	% of pregnant women that used hospital for c/s (*n* = 294)	21	7.1	6	5.1	85	>15% excess 5–15% acceptable <5% low	Acceptable
	*Total compliance score and judgment*			*29*	*13.6*	*46.9*		*critical*

**Table 6:** Acceptability dimension with selected indicators and judgment, Hadiya zone, SNNPR, Ethiopia, April 2014.

S. no.	Indicator	Accepted		Expected weight of indicator	Gained weight of indicator	%	Judgment criteria and judgment	Judgment of indicator
		Number	%					
1	% of mothers accepted cost of skilled delivery service (*n* = 294).	254	86.4	6	5.184	86.4	>85% adequate	Adequate
2	% of mothers happy with the presence of 24 hr skilled birth service (*n* = 294)	293	99.7	4	3.988	99.7	70–85% acceptable	Adequate
3	% of mother accepted health facilities skilled delivery service (*n* = 294)	273	92.9	4	3.716	92.9		Adequate
4	% of mothers with families, who is accepted the health facilities delivery service for future (*n* = 846)	764	90.3	6	5.418	90.3	50–69.9% partial	Adequate
5	Proportion of mothers happy with the care of herself during health facilities skilled delivery service (*n* = 294)	285	96.9	6	5.814	96.9	<50% critical	Adequate
6	% of mothers happy with the care of the newborn during health facilities skilled delivery service (*n* = 294)	293	99.7	6	5.982	99.7		Adequate
7	% of mothers happy with the appearance of the delivery room (*n* = 294)	292	99.3	2	1.986	99.3		Adequate
8	% of mothers happy with the processes and procedures during health facilities skilled delivery service (*n* = 294)	273	92.8	6	5.568	92.8		Adequate
9	% of mothers happy with the sex of skilled birth attendant (*n* = 294)	290	98.6	5	4.93	98.6		Adequate
10	% of mothers happy with the age of birth attendants (*n* = 294)	293	99.7	4	3.988	99.7		Adequate
11	% of mothers happy with the waiting time to get service (*n* = 294)	289	98.2	4	3.928	98.2		Adequate
12	% of mothers happy with the birth attendants help and supportiveness (*n* = 294)	262	89.1	5	4.455	89.1		Adequate
13	% of mothers happy with the birth attendant's response to theire question (*n* = 294)	258	87.7	4	3.508	87.7		Adequate
14	% of mothers happy with the privacy keeping in waiting and delivery room (*n* = 294)	282	95.9	4	3.836	95.9		Adequate
15	% of mothers happy with the comfortability of waiting and delivery room (*n* = 294)	293	99.6	2	1.992	99.6		Adequate
16	% of mothers happy with the distance from home to health facility (*n* = 294)	285	96.9	4	3.876	96.9		Adequate
	*Total score and judgment of acceptability*			*72*	*68.169*	*94.68*		*Adequate*

## Data Availability

The data used to support the findings of this study are available from the corresponding author upon request.

## Abbreviations

ANC: Antenatal care
C/S: Cesarean section
EDHS: Ethiopian Demographic and Health Survey
EFY: Ethiopian financial year
FP: Family planning
HSDP: Health Sector Development Program
KM: Kilo meter
MDG: Millennium Development Goal
PNC: Postnatal care
SBA: Skilled birth attendant
SNNPR: Southern Nations, Nationalities, and Peoples Region
SPSS: Statistical Package for Social Science
UN: United Nation
WHO: World Health Organization.
